# Supplementary material for: Linking the evolution of two prefrontal brain regions to social and foraging challenges in primates
Source: eLife. 2024 Oct 29;12:RP87780. doi: 10.7554/eLife.87780 (PMC11521368; doi:10.7554/eLife.87780)
Supplement: Supplementary file 1. — AIC values have been computed for a regression model without (OLS) and with (phylogenetic generalized least squares [PGLS]) phylogenetic correlation. The smallest value for each brain region (response) is in bold. [file elife-87780-supp1.docx]

| **Response** | **Predictor(s)** | **OLS** | **PGLS** |
| --- | --- | --- | --- |
| WB | log10(Body) | 1.31 | 12.47 |
|  | Pop_d | 16.82 | 14.12 |
|  | DTD | 10.18 | 3.47 |
|  | log10(Body) + Pop_d | 0.51 | 8.33 |
|  | log10(Body) + DTD | -7.20 | 0.50 |
|  | Pop_d + DTD | 11.08 | 3.85 |
|  | log10(Body) + Pop_d + DTD | **-8.61** | **-3.57** |
| FP | log10(Body) | 18.08 | 25.06 |
|  | Pop_d | 31.38 | 25.57 |
|  | DTD | 26.78 | 17.16 |
|  | log10(Body) + Pop_d | 17.59 | 19.78 |
|  | log10(Body) + DTD | 14.83 | 15.23 |
|  | Pop_d + DTD | 28.02 | 16.74 |
|  | log10(Body) + Pop_d + DTD | **14.47** | **9.99** |
| DLPFC | log10(Body) | 4.08 | 12.90 |
|  | Pop_d | 20.75 | 14.50 |
|  | DTD | 14.96 | 5.78 |
|  | log10(Body) + Pop_d | 3.39 | 9.37 |
|  | log10(Body) + DTD | -3.61 | 3.67 |
|  | Pop_d + DTD | 15.66 | 6.44 |
|  | log10(Body) + Pop_d + DTD | **-4.72** | **1.01** |
