# Supplementary material for: Linking the evolution of two prefrontal brain regions to social and foraging challenges in primates
Source: eLife. 2024 Oct 29;12:RP87780. doi: 10.7554/eLife.87780 (PMC11521368; doi:10.7554/eLife.87780)
Supplement: Supplementary file 2. — This table indicates the values of the normalized coefficients for each of the brain region and each of the variables of the regression models. [file elife-87780-supp2.docx]

| **Response** | **Predictors** | | |
| --- | --- | --- | --- |
|  | **log10(Body)** | **Pop_d** | **DTD** |
| WB | 0.71 | 0.35 | 0.42 |
| FP (exc. dorsal area) | 0.66 | 0.38 | 0.36 |
| FP (inc. dorsal area) | 0.55 | 0.37 | 0.52 |
| DLPFC | 0.62 | 0.30 | 0.35 |
