# Supplementary material for: Linking the evolution of two prefrontal brain regions to social and foraging challenges in primates
Source: eLife. 2024 Oct 29;12:RP87780. doi: 10.7554/eLife.87780 (PMC11521368; doi:10.7554/eLife.87780)
Supplement: Supplementary file 4. — For 10 species (i.e. the monkeys species), the volume of FP was re-assessed as described in the text, because the delimitation of the FP could be considered from a different perspective. This procedure was not necessary for great apes and humans, because in those species the delimitation of FP was easier. In short, we used a conservative measure of the FP in monkeys, which did not include the dorsal part of the anterior prefrontal cortex. Indeed, even anatomo-functional studies suggest that the the FP could extend more dorsally in macaques, that dorsal region also includes Brodmann area 9 (BA 9), which is usually associated with the DLPFC (Sallet et al., 2013; Petrides, 2005. Petrides et al., 2012). But since that dorsal area could also be associated with the FP, we conducted a new analysis where the FP volume did include that dorsal area. Fig SF 4.1. Relation between conservative and inclusive measures of the FP volume Table SF 4.2: Model comparison data for the inclusive measure of the FP. Each line corresponds to a model tested to account for the variability of FP volumes across species. Each model is defined as a set of predictors. We provide the AIC of each model using both OLS and PGLS regression methods. For each method, the AIC value of the best model (smallest AIC value) is indicated in bold. Table SI 4.3. Parameter estimate for the best model according to OLS. The table provides the mean beta estimate, its standard error (SE), as well as the corresponding t statistic and p-value estimate for each of the parameters of the best model based on OLS approach (i.e. AIC = 10.22). Table S4.4. Parameter estimate for the best model according to PGLS. The table provides the mean beta estimate, its SE, as well as the corresponding t statistic and p value estimate for each of the parameters of the best model based on PGLS approach (i.e. AIC = 10.25). [file elife-87780-supp4.docx]

*Fig SF 4.1: Relation between conservative and inclusive measures of the FP volume*


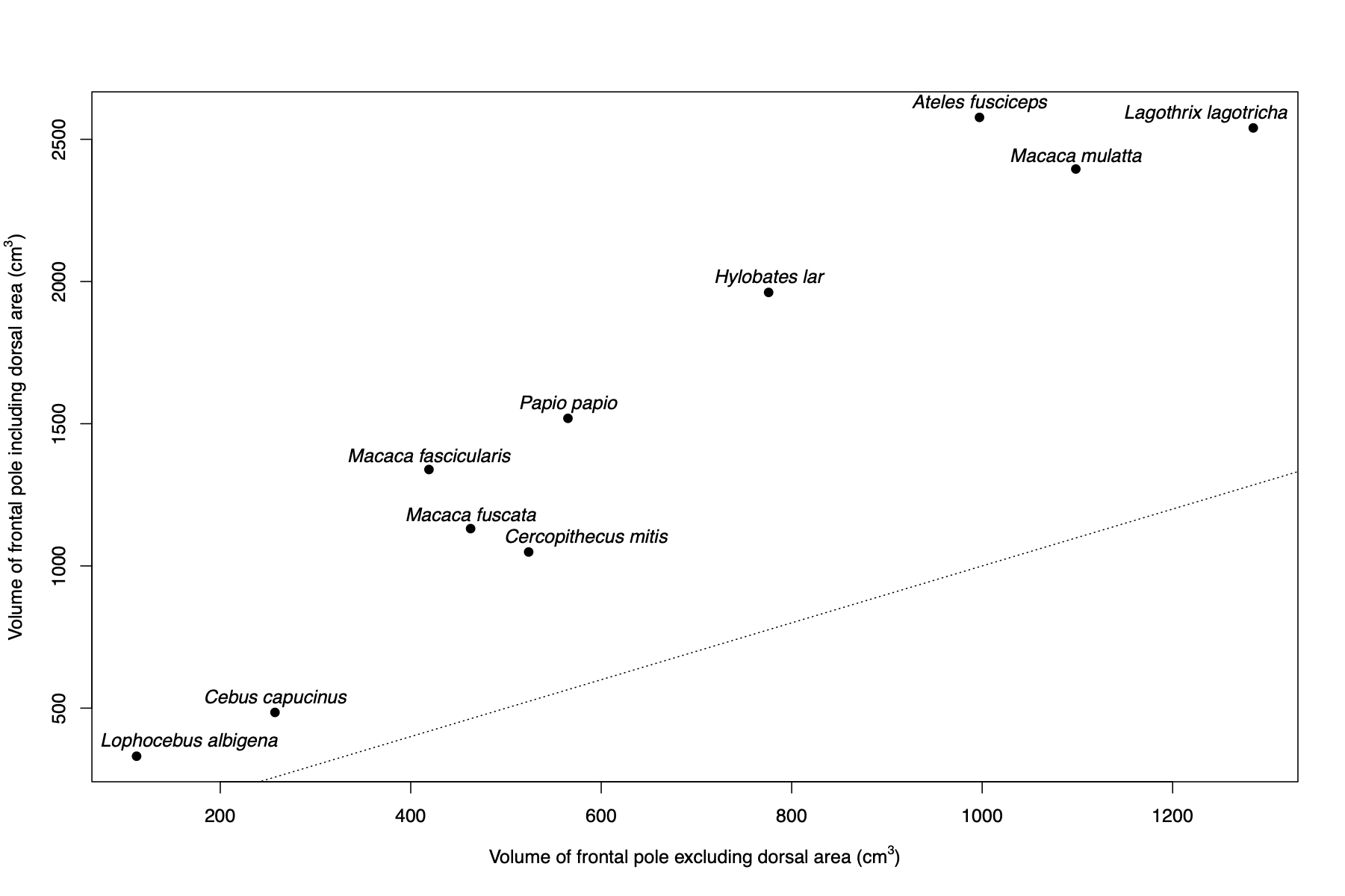


*Table SF 4.2: Model comparison data for the inclusive measure of the FP*

| **Response** | **Predictor(s)** | **OLS** | **PGLS** |
| --- | --- | --- | --- |
| FP (inc. dorsal area) | log10(Body) | 15.19 | 23.95 |
| FP (inc. dorsal area) | Pop_d | 22.54 | 23.27 |
| FP (inc. dorsal area) | DTD | 15.14 | 12.18 |
| FP (inc. dorsal area) | log10(Body) + Pop_d | 15.23 | 20.98 |
| FP (inc. dorsal area) | log10(Body) + DTD | **10.22** | 12.45 |
| FP (inc. dorsal area) | Pop_d + DTD | 16.98 | 12.18 |
| FP (inc. dorsal area) | log10(Body) + Pop_d + DTD | 10.37 | **10.25** |

*Table SI 4.3: Parameter estimate for the best model according to OLS*

| **Coefficient** | **Estimate** | **SE** | ***t*-test** | ***P*** |
| --- | --- | --- | --- | --- |
| (intercept) | 2.660 | 0.182 | 14.623 | 0 |
| log10(Body) | 0.376 | 0.142 | 2.653 | 0.020 |
| DTD | 0.067 | 0.025 | 2.663 | 0.019 |

*Table SI 4.4: Parameter estimate for the best model according to PGLS*

| **Coefficient** | **Estimate** | **SE** | ***t*-test** | ***P*** |
| --- | --- | --- | --- | --- |
| (intercept) | 2.455 | 0.371 | 6.610 | 0 |
| log10(Body) | 0.430 | 0.235 | 1.830 | 0.092 |
| Pop_d | 0.008 | 0.004 | 1.900 | 0.081 |
| DTD | 0.072 | 0.019 | 3.821 | 0.002 |
