# Supplementary material for: Linking the evolution of two prefrontal brain regions to social and foraging challenges in primates
Source: eLife. 2024 Oct 29;12:RP87780. doi: 10.7554/eLife.87780 (PMC11521368; doi:10.7554/eLife.87780)
Supplement: Supplementary file 5. — Each of the three panels corresponds to one of the variables of the model, and shows the value (colored dots) of the estimated coefficient for that variable after the data of the corresponding species has been removed from the analysis. The color code is indicated on the figure, below Pop_d panel. As a reminder, we added the values of these estimated coefficients (together with t and p statistics) when all species are included. The values of these ‘original’ coefficients (i.e. when all species are included) are indicated by a vertical dotted line on the panel of each of these variables. Body: body weight; DTD: daily traveled distance; Pop_d: population density. [file elife-87780-supp5.pdf]

Response: FP

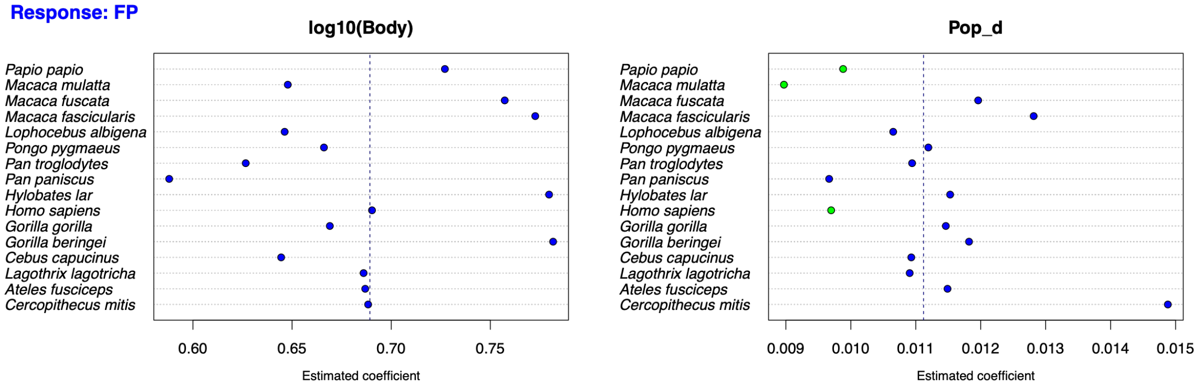

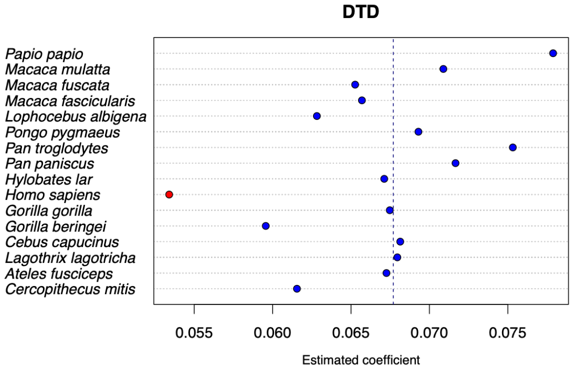

Each row shows the value of the coefficient after removing the species.

- $P > 0.1$ (red)
- $0.05 \le P < 0.1$ (green)
- $P < 0.05$ (blue)

The vertical dashed lines show the coefficients with all species.

MODEL WITH ALL SPECIES

	**Value**	**Std.Error**	**t–value**	**p–value**
**(Intercept)**	1.869	0.368	5.075	0
**log10(Body)**	0.689	0.233	2.955	0.012
**Pop_d**	0.011	0.004	2.62	0.022
**DTD**	0.068	0.019	3.615	0.004
