# Supplementary material for: Linking the evolution of two prefrontal brain regions to social and foraging challenges in primates
Source: eLife. 2024 Oct 29;12:RP87780. doi: 10.7554/eLife.87780 (PMC11521368; doi:10.7554/eLife.87780)
Supplement: Supplementary file 7. — There are six continuous and five categorical variables. Table SF7.1. Shows pairwise correlations for all pairs of continuous variables (Pearson correlation coefficients below the diagonal; p-values above the diagonal). If we consider a Bonferroni correction for these tests, the critical p-value becomes 0.05/3=0.003. Legends: Body for body mass; QI for dietary quality index; DTD for daily traveled distance; Gp for group size; Pop_d for population density; weaning for ratio weaning period/lifespan. Fig SF 7.2. Shows Pairwise plots of correlations across these continuous socio-ecological variables. Table SF 7.3. Contingency table of the two correlated categorical variables: social system and mating system. Among the five categorical variables, only one pair was statistically significant (Fisher test, p=0.0009): social system and mating system. These two variables are actually mostly redundant as shown by their contingency table. Fig SF 7.4. Relations between continuous vs categorical variables. When looking at the relationships between a continuous and a categorical variables, social system appeared as related to most continuous variables, especially body mass and weaning. Table SF 7.5. Quantification of the relation between continuous vs categorical variables. We performed an ANOVA on these 30 pairs with the continuous variable as response. Setting the significance threshold at 0.05/30=0.0017, only one fit was statistically significant: social system on body mass. If the response (continuous variable) was log-transformed, only one analysis was significant: social system on group size. [file elife-87780-supp7.docx]

*Table SF 7.1:* Pairwise correlations for all pairs of continuous variables

|  | Body | DQI | DTD | Gp | Pop_d | Weaning |
| --- | --- | --- | --- | --- | --- | --- |
| Body |  | 0.011 | 0.867 | 0.780 | 0.067 | 0.006 |
| DQI | -0.617 |  | 0.063 | 0.266 | 0.159 | 0.019 |
| DTD | 0.046 | 0.475 |  | 0.000 | 0.891 | 0.292 |
| Gp | -0.076 | 0.296 | 0.822 |  | 0.870 | 0.270 |
| Pop_d | -0.469 | 0.370 | -0.037 | -0.045 |  | 0.022 |
| Weaning | 0.652 | -0.577 | -0.281 | -0.293 | -0.566 |  |

*Fig SF 7.2:* Pairwise plots of correlations across continuous socio-ecological variables.
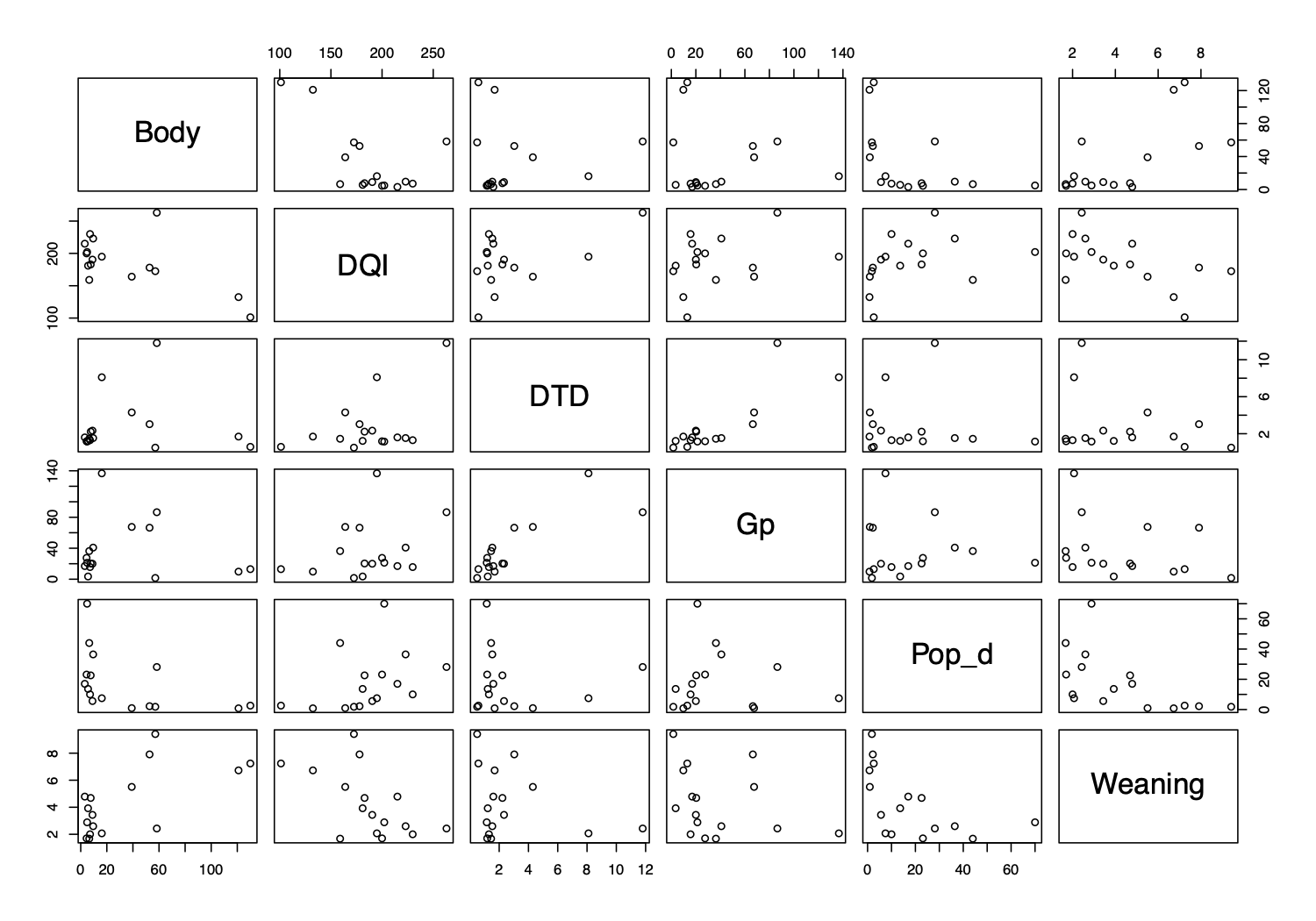


Table SF 7.3: Contingency table of the 2 correlated categorical variables: Social System and Mating System.

|  | Harem polygyny | Monogamy | Polygynandry | Spatial polygyny |
| --- | --- | --- | --- | --- |
| Multi-male multi-female | 3 | 0 | 9 | 0 |
| Pair | 0 | 1 | 0 | 0 |
| Single male | 2 | 0 | 0 | 0 |
| Solitary | 0 | 0 | 0 | 1 |

*Fig SF 7.4: Boxplots of relation between continuous vs categorical variables
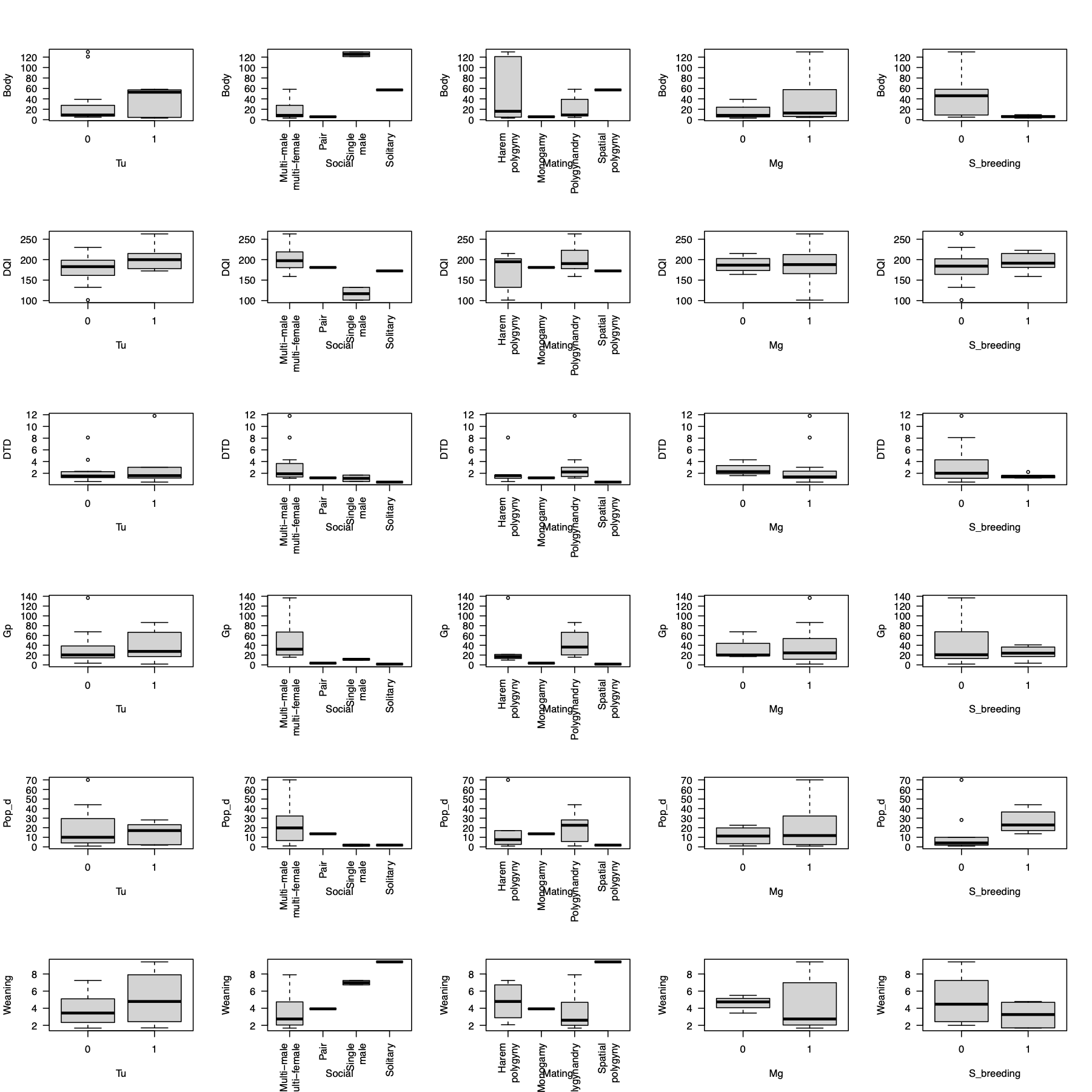
*

*Table SI 7.5: Quantification of the relation between continus vs categorical variables.*

| **Continuous Var.** | **Categorical Var.** | **P-value** | **P-value_log(Cont. V)** |
| --- | --- | --- | --- |
| Body | Tool Use (tu) | 0.9066 | 0.7374 |
| Body | Social | 0.0001 | 0.0210 |
| Body | Mating | 0.4435 | 0.5965 |
| Body | Mate guarding (Mg) | 0.3146 | 0.3534 |
| Body | S_breeding | 0.0361 | 0.0057 |
| DQI | Tool Use (tu) | 0.1953 | 0.2133 |
| DQI | Social | 0.0191 | 0.0041 |
| DQI | Mating | 0.5952 | 0.5491 |
| DQI | Mate guarding (Mg) | 0.9437 | 0.8144 |
| DQI | S_breeding | 0.6095 | 0.5050 |
| DTD | Tool Use (tu) | 0.4575 | 0.8264 |
| DTD | Social | 0.6578 | 0.1643 |
| DTD | Mating | 0.8248 | 0.3263 |
| DTD | Mate guarding (Mg) | 0.9220 | 0.4887 |
| DTD | S_breeding | 0.2270 | 0.4255 |
| Gp | Tool Use (tu) | 0.8142 | 0.9169 |
| Gp | Social | 0.3294 | 0.0014 |
| Gp | Mating | 0.6116 | 0.0063 |
| Gp | Mate guarding (Mg) | 0.7476 | 0.7579 |
| Gp | S_breeding | 0.3103 | 0.7023 |
| Pop_d | Tool Use (tu) | 0.6456 | 0.9510 |
| Pop_d | Social | 0.4628 | 0.1187 |
| Pop_d | Mating | 0.8709 | 0.6741 |
| Pop_d | Mate guarding (Mg) | 0.4642 | 0.6738 |
| Pop_d | S_breeding | 0.1942 | 0.0201 |
| Weaning | Tool Use (tu) | 0.3157 | 0.5044 |
| Weaning | Social | 0.0189 | 0.0719 |
| Weaning | Mating | 0.1299 | 0.2857 |
| Weaning | Mate guarding (Mg) | 0.7920 | 0.4331 |
| Weaning | S_breeding | 0.1758 | 0.2188 |
