# Supplementary material for: Linking the evolution of two prefrontal brain regions to social and foraging challenges in primates
Source: eLife. 2024 Oct 29;12:RP87780. doi: 10.7554/eLife.87780 (PMC11521368; doi:10.7554/eLife.87780)
Supplement: Supplementary file 8. — Table SF 8.1. Socio-ecological variables. This table indicates the socio-ecological variables of interest for each of the species. BM: body mass (kg); DQI: dietary quality index; TU: tool use (yes/no); DTD: daily traveled distance (km); GS: group size; Pop D: population density (ind/km2); Soc. System: social system; MG: mate guarding (yes/no); Seas. B: seasonal breading (yes/no); Mating syst: Mating system. WL: ratio weaning period/lifespan. Table SF 8.2. References socio-ecological variables. This table shows the references used to calculate the values of each socio-ecological variable of each species. Only names and dates of publication are provided in the table, but detailed references are provided in the list below. [file elife-87780-supp8.docx]

*Table SF 8.1: socio-ecological variables*

| **Species** | **BM** | **DQI** | **TU** | **DTD** | **GS** | **Pop D** | **Soc. System** | **MG** | **Seas. B** | **Mating Syst** | **WL** |
| --- | --- | --- | --- | --- | --- | --- | --- | --- | --- | --- | --- |
| *Ateles fusciceps* | 9.0 | 190.4 | 0 | 2.3 | 20.0 | 5.6 | Polygynandry | 0 | 0 | Polygynandry | 3.4 |
| *Cebus capucinus* | 3.1 | 215.0 | 1 | 1.6 | 17.0 | 17.0 | Polygynandry | 0 | 1 | Harem polygyny | 4.8 |
| *Cercopithecus mitis* | 4.9 | 202.2 | 0 | 1.15 | 21.3 | 70.0 | Polygynandry | 0 | 0 | Harem polygyny | 2.9 |
| *Gorilla gorilla* | 120.9 | 132.4 | 0 | 1.7 | 9.8 | 0.8 | Polygyny | 1 | 0 | Harem polygyny | 6.7 |
| *Gorilla beringei* | 130.0 | 101.2 | 0 | 0.6 | 13.0 | 2.6 | Polygyny | 1 | 0 | Harem polygyny | 7.2 |
| *Homo sapiens* | 58.3 | 263.0 | 1 | 11.8 | 86.5 | 28.1 | Polygynandry | 1 | 0 | Polygynandry | 2.4 |
| *Hylobates lar* | 5.6 | 181.0 | 0 | 1.2 | 3.5 | 13.6 | Pair | 1 | 1 | Monogamy | 3.9 |
| *Lagothrix lagotricha* | 7.7 | 182.9 | 0 | 2.2 | 20.3 | 22.6 | Polygynandry | 0 | 1 | Polygynandry | 4.7 |
| *Lophocebus albigena* | 7.1 | 229.9 | 0 | 1.3 | 15.7 | 10.0 | Polygynandry | 1 | 0 | Polygynandry | 2,0 |
| *Macaca fascicularis* | 4.5 | 200.0 | 1 | 1.2 | 27.5 | 23.1 | Polygynandry | 1 | 1 | Polygynandry | 1.7 |
| *Macaca fuscata* | 9.5 | 223.0 | 0 | 1.5 | 40.8 | 36.5 | Polygynandry | 1 | 1 | Polygynandry | 2.6 |
| *Macaca mulatta* | 6.5 | 159.0 | 0 | 1.5 | 36.3 | 44.0 | Polygynandry | 1 | 1 | Polygynandry | 1.7 |
| *Pan troglodytes* | 52.8 | 178.0 | 1 | 3.0 | 66.5 | 2.2 | Polygynandry | 1 | 0 | Polygynandry | 7.9 |
| *Pan paniscus* | 39.1 | 164.0 | 0 | 4.3 | 67.5 | 0.9 | Polygynandry | 0 | 0 | Polygynandry | 5.5 |
| *Papio papio* | 16.2 | 194.9 | 0 | 8.1 | 136.6 | 7.5 | Polygynandry | 1 | 0 | Harem polygyny | 2.1 |
| *Pongo pygmaeus* | 57.2 | 172.5 | 1 | 0.5 | 1.6 | 1.8 | Solitary | 1 | 0 | Spatial polygyny | 9.4 |

*Table SF 8.2: References for socio-ecological data
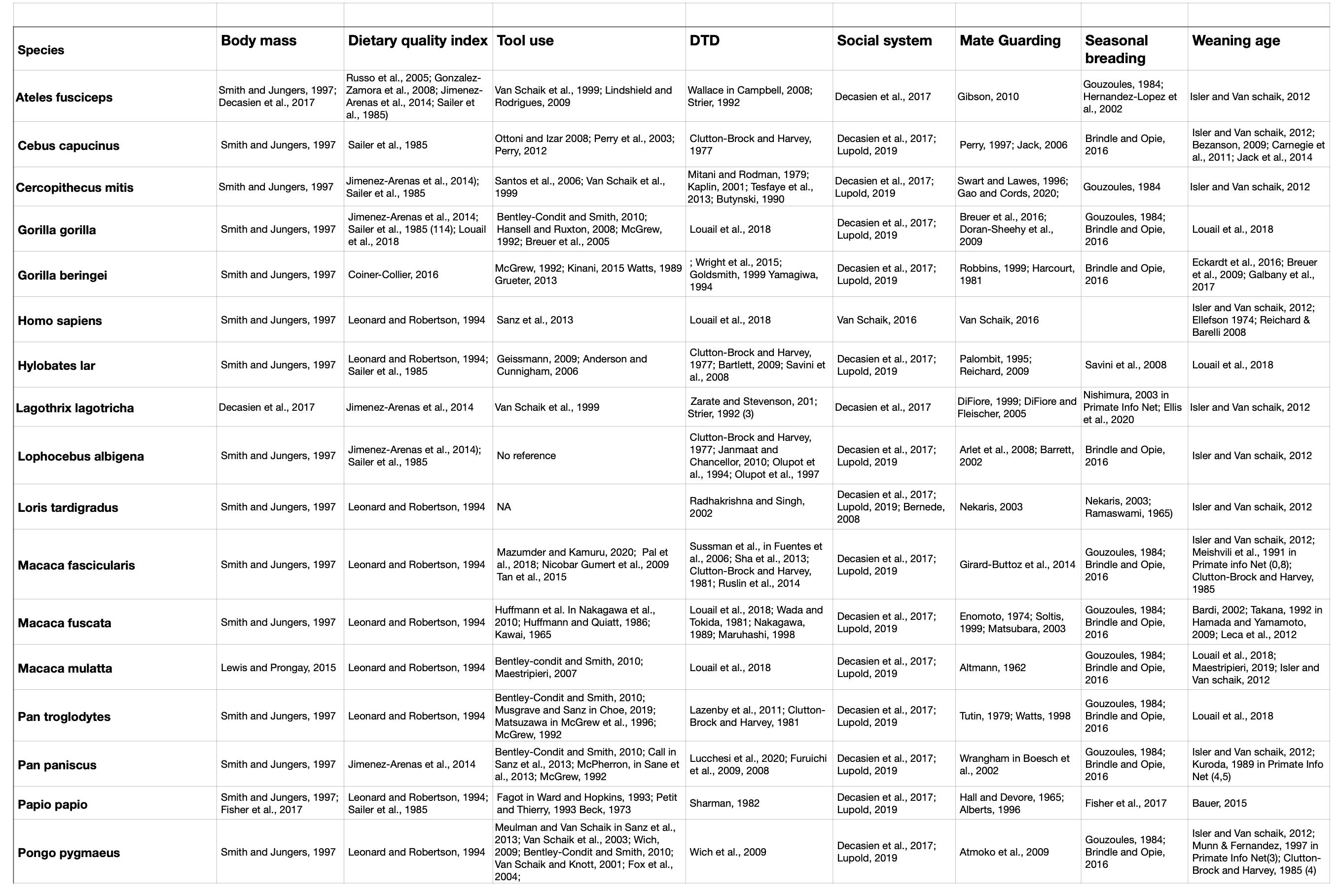
*

***References list:***

Alberts, Susan C., Jeanne Altmann, and Michael L. Wilson. 1996. 'Mate guarding constrains foraging activity of male baboons', *Animal Behaviour*, 51: 1269-77.

Altmann, Stuart A. 1962. 'A Field Study of the Sociobiology of Rhesus Monkeys, Macaca Mulatta *', *Annals of the New York Academy of Sciences*, 102: 338-435.

Arlet, Małgorzata E., Freerk Molleman, and Colin A. Chapman. 2008. 'Mating Tactics in Male Grey-Cheeked Mangabeys (Lophocebus albigena)', *Ethology*, 114: 851-62.

Atmoko, SS Utami, T Mitra Setia, Benoit Goossens, Sheena S James, Cheryl D Knott, Helen C Morrogh-Bernard, Carel P van Schaik, and Maria A van Noordwijk. 2009. 'Orangutan mating behavior and strategies', *Orangutans: Geographic variation in behavioral ecology and conservation*: 235-44.

Bardi, Massimo, and Michael A Huffman. 2002. 'Effects of maternal style on infant behavior in Japanese macaques (Macaca fuscata)', *Developmental Psychobiology: The Journal of the International Society for Developmental Psychobiology*, 41: 364-72.

Barrett, Gordon M., Keiko Shimizu, Massimo Bardi, Shinsuke Asaba, and Akio Mori. 2002. 'Endocrine Correlates of Rank, Reproduction, and Female-Directed Aggression in Male Japanese Macaques (Macaca fuscata)', *Hormones and Behavior*, 42: 85-96.

Bartlett, Thad Q. 2009. 'Seasonal Home Range Use and Defendability in White-Handed Gibbons (Hylobates lar) in Khao Yai National Park, Thailand.' in, *The Gibbons*.

Bauer, Cassondra. 2015. 'The baboon (Papio sp.) as a model for female reproduction studies', *Contraception*, 92: 120-23.

Beck, Benjamin B. 1973. 'Observation learning of tool use by captive Guinea baboons (Papio papio)', *American Journal of Physical Anthropology*, 38: 579-82.

Bentley-Condit, Vicki, and Smith. 2010. 'Animal tool use: current definitions and an updated comprehensive catalog', *Behaviour*, 147: 185-32A.

Bezanson, Michelle. 2009. 'Life history and locomotion in Cebus capucinus and Alouatta palliata', American Journal of Physical Anthropology: The Official Publication of the American Association of Physical Anthropologists, 140: 508-17.

Boesch, Christophe, Gottfried Hohmann, and Linda F. Marchant. 2002. *Behavioural diversity in chimpanzees and bonobos* (Cambridge University Press: Cambridge).

Breuer, Thomas, Mireille Breuer‐Ndoundou Hockemba, Claudia Olejniczak, Richard J Parnell, and Emma J Stokes. 2009. 'Physical maturation, life‐history classes and age estimates of free‐ranging western gorillas—Insights from Mbeli Bai, Republic of Congo', *American Journal of Primatology: Official Journal of the American Society of Primatologists*, 71: 106-19.

Breuer, Thomas, Andrew M. Robbins, and Martha M. Robbins. 2016. 'Sexual coercion and courtship by male western gorillas', *Primates*, 57: 29-38.

Brindle, Matilda, and Christopher Opie. 2016. 'Postcopulatory sexual selection influences baculum evolution in primates and carnivores', *Proceedings of the Royal Society B: Biological Sciences*, 283: 20161736.

Butynski, Thomas M. 1990. 'Comparative ecology of blue monkeys (Cercopithecus mitis) in high‐and low‐density subpopulations', *Ecological Monographs*, 60: 1-26.

Campbell, Christina J. 2017. 'Primates in Perspective.' in, *The International Encyclopedia of Primatology*.

Carnegie, Sarah D, Linda M Fedigan, and Amanda D Melin. 2011. 'Reproductive seasonality in female capuchins (Cebus capucinus) in Santa Rosa (Area de Conservación Guanacaste), Costa Rica', *International Journal of Primatology*, 32: 1076-90.

Choe, Jae C. 2019. *Encyclopedia of animal behavior* (Elsevier/Academic Press: Amsterdam).

Clutton‐Brock, T. H., and Paul H. Harvey. 1977. 'Primate ecology and social organization', *Journal of Zoology*, 183: 1-39.

Coiner-Collier, S., R. S. Scott, J. Chalk-Wilayto, S. M. Cheyne, P. Constantino, N. J. Dominy, A. A. Elgart, H. Glowacka, L. C. Loyola, K. Ossi-Lupo, M. Raguet-Schofield, M. G. Talebi, E. A. Sala, P. Sieradzy, A. B. Taylor, C. J. Vinyard, B. W. Wright, N. Yamashita, P. W. Lucas, and E. R. Vogel. 2016. 'Primate dietary ecology in the context of food mechanical properties', *J Hum Evol*, 98: 103-18.

Cunningham, Clare L., James R. Anderson, and Alan R. Mootnick. 2006. 'Object manipulation to obtain a food reward in hoolock gibbons, Bunopithecus hoolock', *Animal Behaviour*, 71: 621-29.

DeCasien, Alex R., Scott A. Williams, and James P. Higham. 2017. 'Primate brain size is predicted by diet but not sociality', *Nature Publishing Group*, 1: 1-7.

Doran-Sheehy, Diane M., David Fernández, and Carola Borries. 2009. 'The strategic use of sex in wild female western gorillas', *American Journal of Primatology*, 71: 1011-20.

Eckardt, Winnie, Katie Fawcett, and Alison W Fletcher. 2016. 'Weaned age variation in the Virunga mountain gorillas (Gorilla beringei beringei): influential factors', *Behavioral Ecology and Sociobiology*, 70: 493-507.

Ellis, Kelsey M, Laura A Abondano, Andrés Montes‐Rojas, Andrés Link, and Anthony Di Fiore. 2021. 'Reproductive seasonality in two sympatric primates (Ateles belzebuth and Lagothrix lagotricha poeppigii) from Amazonian Ecuador', *American Journal of Primatology*, 83: e23220.

Fiore, Anthony Di, and Robert C. Fleischer. 2005. 'Social Behavior, Reproductive Strategies, and Population Genetic Structure of Lagothrix poeppigii', *International Journal of Primatology*, 26: 1137-73.

Fischer, Julia, Gisela H. Kopp, Federica Dal Pesco, Adeelia Goffe, Kurt Hammerschmidt, Urs Kalbitzer, Matthias Klapproth, Peter Maciej, Ibrahima Ndao, Annika Patzelt, and Dietmar Zinner. 2017. 'Charting the neglected West: The social system of Guinea baboons', *American Journal of Physical Anthropology*, 162: 15-31.

Fox, ElizaBeth A., Carel P. van Schaik, Arnold Sitompul, and Donielle N. Wright. 2004. 'Intra-and interpopulational differences in orangutan (Pongo pygmaeus) activity and diet: Implications for the invention of tool use', *American Journal of Physical Anthropology*, 125: 162-74.

Furuichi, Takeshi. 2009. 'Factors underlying party size differences between chimpanzees and bonobos: a review and hypotheses for future study', *Primates*, 50: 197-209.

Galbany, Jordi, Didier Abavandimwe, Meagan Vakiener, Winnie Eckardt, Antoine Mudakikwa, Felix Ndagijimana, Tara S Stoinski, and Shannon C McFarlin. 2017. 'Body growth and life history in wild mountain gorillas (Gorilla beringei beringei) from Volcanoes National Park, Rwanda', *American Journal of Physical Anthropology*, 163: 570-90.

Gao, Lu, and Marina Cords. 2020. 'Effects of Female Group Size on the Number of Males in Blue Monkey (Cercopithecus mitis) Groups', *International Journal of Primatology*, 41: 665-82.

Geissmann, Thomas. 2009. "Door slamming: Tool-use by a captive white-handed gibbon (Hylobates lar)." In.

Gibson, K. Nicole. 2010. 'Male mating tactics in spider monkeys: sneaking to compete', *American Journal of Primatology*, 72: 794-804.

Girard-Buttoz, Cédric, Michael Heistermann, Erdiansyah Rahmi, Muhammad Agil, Panji Ahmad Fauzan, and Antje Engelhardt. 2014. 'Costs of mate-guarding in wild male long-tailed macaques (Macaca fascicularis): Physiological stress and aggression', *Hormones and Behavior*, 66: 637-48.

Goldsmith, Michele L. 1999. 'Ecological Constraints on the Foraging Effort of Western Gorillas (Gorilla gorilla gorilla) at Bai Hoköu, Central African Republic', *International Journal of Primatology*, 20: 1-23.

González-Zamora, Arturo, Víctor Arroyo-Rodríguez, Óscar M. Chaves, Sonia Sánchez-López, Kathryn E. Stoner, and Pablo Riba-Hernández. 2009. 'Diet of spider monkeys (Ateles geoffroyi) in Mesoamerica: current knowledge and future directions', *American Journal of Primatology*, 71: 8-20.

Gouzoules, Sarah. 1984. 'Primate mating systems, kin associations, and cooperative behavior: Evidence for kin recognition?', *American Journal of Physical Anthropology*, 27: 99-134.

Grueter, Cyril C., Martha M. Robbins, Felix Ndagijimana, and Tara S. Stoinski. 2013. 'Possible tool use in a mountain gorilla', *Behavioural Processes*, 100: 160-62.

Gumert, Michael D., Marius Kluck, and Suchinda Malaivijitnond. 2009. 'The physical characteristics and usage patterns of stone axe and pounding hammers used by long-tailed macaques in the Andaman Sea region of Thailand', *American Journal of Primatology*, 71: 594-608.

Hall, K. R. L., & I. DeVore. 1965. 'Baboon social behavior.' in Ed. I. DeVore (ed.), *Primate Behavior: Field Studies of Monkeys and Apes.* (New York, London:).

Hansell, M., and G. Ruxton. 2008. 'Setting tool use within the context of animal construction behaviour', *Trends in Ecology & Evolution*, 23: 73-78.

Harcourt, Alexander H, and Kelly J Stewart. 1981. 'Gorilla male relationships: Can differences during immaturity lead to contrasting reproductive tactics in adulthood?', *Animal Behaviour*, 29: 206-10.

Harvey, Paul H., and T. H. Clutton-Brock. 1981. 'Primate home-range size and metabolic needs', *Behavioral Ecology and Sociobiology*, 8: 151-55.

Hernández‐López, Leonor, Gerardo Cerezo Parra, Ana Lilia Cerda‐Molina, Stephanella C Pérez‐Bolaños, Vicente Díaz Sánchez, and Ricardo Mondragón‐Ceballos. 2002. 'Sperm quality differences between the rainy and dry seasons in captive black‐handed spider monkeys (Ateles geoffroyi)', *American Journal of Primatology: Official Journal of the American Society of Primatologists*, 57: 35-41.

Huffman, Michael A., and Duane Quiatt. 1986. 'Stone handling by Japanese macaques (Macaca fuscata): Implications for tool use of stone', *Primates*, 27: 413-23.

Isler, Karin, and Carel P van Schaik. 2012. 'Allomaternal care, life history and brain size evolution in mammals', *Journal of human evolution*, 63: 52-63.

Jack, Katharine M, Valérie AM Schoof, Claire R Sheller, Catherine I Rich, Peter P Klingelhofer, Toni E Ziegler, and Linda Fedigan. 2014. 'Hormonal correlates of male life history stages in wild white-faced capuchin monkeys (Cebus capucinus)', *General and comparative endocrinology*, 195: 58-67.

Jack, Katharine M., and Linda M. Fedigan. 2006. 'Why Be Alpha Male? Dominance and Reproductive Success in Wild White-Faced Capuchins (Cebus capucinus).' in Alejandro Estrada, Paul A. Garber, Mary S. M. Pavelka and LeAndra Luecke (eds.), *New Perspectives in the Study of Mesoamerican Primates: Distribution, Ecology, Behavior, and Conservation* (Springer US: Boston, MA).

Janmaat, Karline R. L., and Rebecca L. Chancellor. 2010. 'Exploring New Areas: How Important is Long-Term Spatial Memory for Mangabey (Lophocebus albigena johnstonii) Foraging Efficiency?', *International Journal of Primatology*, 31: 863-86.

Jimenez-Arenas, J. M., J. A. Perez-Claros, J. C. Aledo, and P. Palmqvist. 2014. 'On the relationships of postcanine tooth size with dietary quality and brain volume in primates: implications for hominin evolution', *Biomed Res Int*, 2014: 406507.

Kaplin, Beth A. 2001. 'Ranging Behavior of Two Species of Guenons (Cercopithecus lhoesti and C. mitis doggetti) in the Nyungwe Forest Reserve, Rwanda', *International Journal of Primatology*, 22: 521-48.

Kawai, Masao. 1965. 'Newly-acquired pre-cultural behavior of the natural troop of Japanese monkeys on Koshima islet', *Primates*, 6: 1-30.

Kinani, Jean-Felix, and Dawn Zimmerman. 2015. 'Tool use for food acquisition in a wild mountain gorilla (Gorilla beringei beringei)', *American Journal of Primatology*, 77: 353-57.

Kuroda, Suehisa. 1989. 'Developmental retardation and behavioral characteristics of pygmy chimpanzees.' in, *Understanding chimpanzees* (Harvard University Press).

Lazenby, Richard A., Matthew M. Skinner, Jean-Jacques Hublin, and Christophe Boesch. 2011. 'Metacarpal trabecular architecture variation in the chimpanzee (Pan troglodytes): Evidence for locomotion and tool-use?', *American Journal of Physical Anthropology*, 144: 215-25.

Leca, Jean-Baptiste, Michael A Huffman, and Paul L Vasey. 2012. The monkeys of stormy mountain: 60 years of primatological research on the Japanese macaques of Arashiyama (Cambridge University Press).

Leonard, W. R., and M. L. Robertson. 1994. 'Evolutionary perspectives on human nutrition: The influence of brain and body size on diet and metabolism', *Am J Hum Biol*, 6: 77-88.

Lewis, Anne D., and Kamm Prongay. 2015. 'Basic Physiology of Macaca mulatta.' in, *The Nonhuman Primate in Nonclinical Drug Development and Safety Assessment*.

Lindshield, Stacy M., and Michelle A. Rodrigues. 2009. 'Tool use in wild spider monkeys (Ateles geoffroyi)', *Primates*, 50: 269-72.

Louail, Margot, Emmanuel Gilissen, Sandrine Prat, Cécile Garcia, and Sebastien Bouret. 2019. 'Refining the ecological brain: Strong relation between the ventromedial prefrontal cortex and feeding ecology in five primate species', *Cortex*, 118: 262-74.

Lucchesi, Stefano, Leveda Cheng, Karline Janmaat, Roger Mundry, Anne Pisor, and Martin Surbeck. 2020. 'Beyond the group: how food, mates, and group size influence intergroup encounters in wild bonobos', *Behavioral Ecology*, 31: 519-32.

Lüpold, Stefan, Leigh W. Simmons, and Cyril C. Grueter. 2019. 'Sexual ornaments but not weapons trade off against testes size in primates', *Proceedings of the Royal Society B: Biological Sciences*, 286.

Maestripieri, Dario. 2018. 'Maternal influences on primate social development', *Behavioral Ecology and Sociobiology*, 72: 130.

Maruhashi, Tamaki, Chiemi Saito, and Naoki Agetsuma. 1998. 'Home range structure and inter-group competition for land of Japanese macaques in evergreen and deciduous forests', *Primates*, 39: 291-301.

Matsubara, Miki. 2003. 'Costs of Mate Guarding and Opportunistic Mating Among Wild Male Japanese Macaques', *International Journal of Primatology*, 24: 1057-75.

Mazumder, Jayashree, and Stefano S. K. Kaburu. 2020. 'Correction to: Object Manipulation and Tool Use in Nicobar Long-Tailed Macaques (Macaca fascicularis umbrosus)', *International Journal of Primatology*, 41: 764-64.

McGrew, WC. 1992. "Tool‐use by free‐ranging chimpanzees: the extent of diversity." In.: Wiley Online Library.

McGrew, William C. 2010. Chimpanzee Material Culture.

Meishvili, Natela, and Valery Chalyan. 1992. 'SOCIAL FACTORS AND MOTHER–INFANT RELATIONSHIPS IN CYNOMOLGUS MACAQUES', *Anthropologie*: 181-85.

Mitani, John C., and Peter S. Rodman. 1979. 'Territoriality: The Relation of Ranging Pattern and Home Range Size to Defendability, with an Analysis of Territoriality among Primate Species', *Behavioral Ecology and Sociobiology*, 5: 241-51.

Nakagawa, Naofumi. 1989. 'Bioenergetics of Japanese monkeys (Macaca fuscata) on Kinkazan Island during winter', *Primates*, 30: 441-60.

Nakagawa, Naofumi, Masayuki Nakamichi, and Hideki Sugiura. 2010. "The Japanese macaques." In *Primatology monographs,*, 1 online resource (xxiii, 402 pages). Tokyo ; New York: Springer,.

Nishimura, Akisato. 2003. 'Reproductive parameters of wild female Lagothrix lagotricha', *International Journal of Primatology*, 24: 707-22.

Olupot, William, Colin A. Chapman, Charles H. Brown, and Peter M. Waser. 1994. 'Mangabey (Cercocebus albigena) population density, group size, and ranging: A twenty-year comparison', *American Journal of Primatology*, 32: 197-205.

Olupot, William, Peter M. Waser, and Colin A. Chapman. 1998. 'Fruit Finding by Mangabeys (Lophocebus albigena): Are Monitoring of Fig Trees and Use of Sympatric Frugivore Calls Possible Strategies?', *International Journal of Primatology*, 19: 339-53.

Ottoni, Eduardo B., and Patrícia Izar. 2008. 'Capuchin monkey tool use: Overview and implications', *Evolutionary Anthropology: Issues, News, and Reviews*, 17: 171-78.

Pal, Arijit, Honnavalli N. Kumara, Partha Sarathi Mishra, Avadhoot D. Velankar, and Mewa Singh. 2017. 'Extractive foraging and tool-aided behaviors in the wild Nicobar long-tailed macaque (Macaca fascicularis umbrosus)', *Primates*, 59: 173-83.

Palombit, Ryne A. 1995. 'Longitudinal patterns of reproduction in wild female siamang (Hylobates syndactylus) and white-handed gibbons (Hylobates lar)', *International Journal of Primatology*, 16: 739-60.

Perry, Susan. 1997. 'Male-Female Social Relationships in Wild White-Faced Capuchins (Cebus Capucinus)', *Behaviour*, 134: 477-510.

———. 2018. 'Correction to: Conformism in the food processing techniques of white-faced capuchin monkeys (Cebus capucinus)', *Animal Cognition*, 21: 821-22.

Perry, Susan, Mary Baker, Linda Fedigan, Julie Gros‐Louis, Katherine Jack, Katherine C MacKinnon, Joseph H Manson, Melissa Panger, Kendra Pyle, and Lisa Rose. 2003. 'Social Conventions in Wild White‐faced Capuchin Monkeys', *Current Anthropology*, 44: 241-68.

Petit, O, and B Thierry. 1993. 'Use of stones in a captive group of Guinea baboons (Papio papio)', *Folia Primatologica*.

Reichard, Ulrich H, and Claudia Barelli. 2008. 'Life history and reproductive strategies of Khao Yai Hylobates lar: implications for social evolution in apes', *International Journal of Primatology*, 29: 823-44.

Reichard, Ulrich H. 2009. 'The Social Organization and Mating System of Khao Yai White-Handed Gibbons: 1992-2006.' in Danielle Whittaker and Susan Lappan (eds.), *The Gibbons: New Perspectives on Small Ape Socioecology and Population Biology* (Springer New York: New York, NY).

Robbins, Martha M. 1999. 'Male mating patterns in wild multimale mountain gorilla groups', *Animal Behaviour*, 57: 1013-20.

Ruslin, Farhani, Ikki Matsuda, and Badrul Munir Md-Zain. 2018. 'The feeding ecology and dietary overlap in two sympatric primate species, the long-tailed macaque (Macaca fascicularis) and dusky langur (Trachypithecus obscurus obscurus), in Malaysia', *Primates*, 60: 41-50.

Russo, Sabrina E., Christina J. Campbell, J. Lawrence Dew, Pablo R. Stevenson, and Scott A. Suarez. 2005. 'A Multi-Forest Comparison of Dietary Preferences and Seed Dispersal by Ateles spp', *International Journal of Primatology*, 26: 1017-37.

Sailer, Lee Douglas, Steven J. C. Gaulin, James S. Boster, and Jeffrey A. Kurland. 1985. 'Measuring the relationship between dietary quality and body size in primates', *Primates*, 26: 14-27.

Santos, Laurie R., Heather M. Pearson, Geertrui M. Spaepen, Fritz Tsao, and Marc D. Hauser. 2005. 'Probing the limits of tool competence: Experiments with two non-tool-using species (Cercopithecus aethiops and Saguinus oedipus)', *Animal Cognition*, 9: 94-109.

Sanz, Crickette M., Josep Call, and Christophe Boesch. 2013. *Tool Use in Animals*.

Savini, Tommaso, Christophe Boesch, and Ulrich H. Reichard. 2008. 'Home-range characteristics and the influence of seasonality on female reproduction in white-handed gibbons (Hylobates lar) at Khao Yai National Park, Thailand', *American Journal of Physical Anthropology*, 135: 1-12.

Sha, John Chih Mun, and Goro Hanya. 2013. 'Diet, activity, habitat use, and ranging of two neighboring groups of food‐enhanced long‐tailed macaques (Macaca fascicularis)', *American Journal of Primatology*, 75: 581-92.

Sharman, Martin John. 1982. 'Feeding, ranging and social organisation of the Guinea baboon', University of St Andrews.

Smith, R. J., and W. L. Jungers. 1997. 'Body mass in comparative primatology', *Journal of human evolution*, 32: 523-59.

Soltis, Joseph. 1999. 'Measuring male-female relationships during the mating season in wild Japanese macaques (Macaca fuscata yakui)', *Primates*, 40: 453-67.

Strier, K. B. 1992. 'Atelinae adaptations: behavioral strategies and ecological constraints', *Am J Phys Anthropol*, 88: 515-24.

Swart, Johan, and M. J. Lawes. 1996. 'The effect of habitat patch connectivity on samango monkey (Cercopithecus mitis) metapopulation persistence', *Ecological Modelling*, 93: 57-74.

Tan, Amanda WY. 2017. 'From play to proficiency: The ontogeny of stone-tool use in coastal-foraging long-tailed macaques (Macaca fascicularis) from a comparative perception-action perspective', *Journal of comparative psychology*, 131: 89.

Tanaka, Ichirou. 1992. 'Three phases of lactation in free-ranging Japanese macaques', *Animal Behaviour*, 44: 129-39.

Tesfaye, Dereje, Peter J. Fashing, Afework Bekele, Addisu Mekonnen, and Anagaw Atickem. 2013. 'Ecological Flexibility in Boutourlini’s Blue Monkeys (Cercopithecus mitis boutourlinii) in Jibat Forest, Ethiopia: A Comparison of Habitat Use, Ranging Behavior, and Diet in Intact and Fragmented Forest', *International Journal of Primatology*, 34: 615-40.

Tutin, Caroline E. G. 1979. 'Mating patterns and reproductive strategies in a community of wild chimpanzees (Pan troglodytes schweinfurthii)', *Behavioral Ecology and Sociobiology*, 6: 29-38.

van Schaik, C. P. 2016. 'The Primate Origins of Human Nature', *American Journal of Human Biology*, 28: 950-51.

van Schaik, Carel P., Robert O. Deaner, and Michelle Y. Merrill. 1999. 'The conditions for tool use in primates: implications for the evolution of material culture', *Journal of human evolution*, 36: 719-41.

van Schaik, Carel P., ElizaBeth A. Fox, and Lorri T. Fechtman. 2003. 'Individual variation in the rate of use of tree-hole tools among wild orang-utans: implications for hominin evolution', *Journal of human evolution*, 44: 11-23.

Wada, Kazuo, and Eishi Tokida. 1981. 'Habitat utilization by wintering Japanese monkeys (macaca fuscata fuscata) in the Shiga heights', *Primates*, 22: 330-48.

Ward, Jeannette P, and William D Hopkins. 1993. Primate laterality: Current behavioral evidence of primate asymmetries (Springer Science & Business Media).

Watts, David P. 1989. 'Ant eating behavior of mountain gorillas', *Primates*, 30: 121-25.

———. 1998. 'Coalitionary mate guarding by male chimpanzees at Ngogo, Kibale National Park, Uganda', *Behavioral Ecology and Sociobiology*, 44: 43-55.

Wich, S. A., R. W. Shumaker, L. Perkins, and H. de Vries. 2009. 'Captive and wild orangutan (Pongosp.) survivorship: a comparison and the influence of management', *American Journal of Primatology*, 71: 680-86.

Wright, E., C. C. Grueter, N. Seiler, D. Abavandimwe, T. S. Stoinski, S. Ortmann, and M. M. Robbins. 2015. 'Energetic responses to variation in food availability in the two mountain gorilla populations (Gorilla beringei beringei)', *Am J Phys Anthropol*, 158: 487-500.

Yamagiwa, Juichi, and Ndunda Mwanza. 1994. 'Day-journey length and daily diet of solitary male gorillas in lowland and highland habitats', *International Journal of Primatology*, 15: 207-24.

Zárate, Diego A., and Pablo R. Stevenson. 2014. 'Behavioral Ecology and Interindividual Distance of Woolly Monkeys (Lagothrix lagothricha) in a Rainforest Fragment in Colombia.' in, *The Woolly Monkey*.
